# X-ray Imaging Investigation on the Gilding Technique of an Ancient Egyptian Taweret Wooden Statuette

**DOI:** 10.3390/jimaging7110229

**Published:** 2021-10-29

**Authors:** Luisa Vigorelli, Alessandro Re, Laura Guidorzi, Tiziana Cavaleri, Paola Buscaglia, Marco Nervo, Federica Facchetti, Matilde Borla, Sabrina Grassini, Alessandro Lo Giudice

**Affiliations:** 1 Dipartimento di Elettronica e Telecomunicazioni, Politecnico di Torino, C.so Duca degli Abruzzi 24, 10129 Torino, Italy; luisa.vigorelli@polito.it; 2 Dipartimento di Fisica, Università di Torino, Via Pietro Giuria 1, 10125 Torino, Italy; laura.guidorzi@unito.it (L.G.); alessandro.logiudice@unito.it (A.L.G.); 3 Sezione di Torino, Istituto Nazionale di Fisica Nucleare, Via Pietro Giuria 1, 10125 Torino, Italy; marco.nervo@centrorestaurovenaria.it; 4 Centro Conservazione e Restauro La Venaria Reale, Piazza della Repubblica, Venaria Reale, 10078 Torino, Italy; tiziana.cavaleri@centrorestaurovenaria.it (T.C.); paola.buscaglia@centrorestaurovenaria.it (P.B.); 5 Dipartimento di Economia, Ingegneria, Società e Impresa, Università della Tuscia, Via Santa Maria in Gradi, 4, 01100 Viterbo, Italy; 6 Fondazione Museo delle Antichità Egizie di Torino, Via Accademia delle Scienze 6, 10123 Torino, Italy; federica.facchetti@museoegizio.it; 7 Soprintendenza ABAP-TO, Piazza San Giovanni 2, 10122 Torino, Italy; matilde.borla@beniculturali.it; 8 Dipartimento di Scienza Applicata e Tecnologia, Politecnico di Torino, C.so Duca degli Abruzzi, 24, 10129 Torino, Italy; sabrina.grassini@polito.it

**Keywords:** cultural heritage, archaeometry, conservation, wooden sculpture, ancient Egypt, tomography, gilding

## Abstract

Diagnostic physical methods are increasingly applied to Cultural Heritage both for scientific investigations and conservation purposes. In particular, the X-ray imaging techniques of computed tomography (CT) and digital radiography (DR) are non-destructive investigation methods to study an object, being able to give information on its inner structure. In this paper, we present the results of the X-ray imaging study on an ancient Egyptian statuette (Late Period 722–30 BCE) belonging to the collection of Museo Egizio in Torino and representing an Egyptian goddess called Taweret, carved on wood and gilded with some colored details. Since few specific studies have been focused on materials and techniques used in Ancient Egypt for gilding, a detailed investigation was started in order to verify the technical features of the decoration in this sculpture. Specifically, DR and CT analyses have been performed at the Centro Conservazione e Restauro “La Venaria Reale” (CCR), with a new high resolution flat-panel detector, that allowed us to perform tomographic analysis reaching a final resolution better than the one achievable with the previous apparatus operating in the CCR.

## 1. Introduction

Since the first X-rays radiograph by Roentgen in 1895 and the first application on archaeological finds in 1898, the importance of this imaging technique in cultural heritage was clear. Hence, after more than a century, the potentialities of X-ray radiography are widely known in the field [1]. Moreover, starting from the 1970s, the introduction of computed tomography (CT) added a new powerful non-destructive X-ray technique for the investigation of 3D artworks [2]. The application of CT was introduced to the art and archaeological fields primarily as a tool for analysis of human and animal remains [3,4,5,6,7,8], and then to study other kinds of objects made with various materials, such as glass, metal, clay, and wood [9,10,11,12,13,14,15,16]. Although portable instruments are the best solution to analyze artworks on-site avoiding the transportation of fragile objects [3,17], in the framework of large restoration centers, where hundreds of important artworks are treated every year, it is useful to install a fixed apparatus. This is the case of the instrument working at the Centro Conservazione e Restauro “La Venaria Reale” (CCR) near Torino, in Italy. Due to the great variety of materials and dimensions of artworks in the cultural heritage field, the apparatus at CCR was purposely customized to be more flexible than medical and industrial CT. Developed in the framework of the neu_ART project involving the National Institute for Nuclear Physics (INFN), the Physics Department of the University of Torino, and CCR [18,19], the basic version was designed to perform, in a short time and with a sufficiently good resolution for restoration purposes, Digital Radiographies (DR) of large objects, up to 4 m × 3 m [20]. Moreover, the DR apparatus was integrated into a more complex system that can be used for the computed tomography of voluminous objects up to 2.5 m in height and 2 m in width [10,21,22].

The necessity at CCR to analyze small objects with a better resolution or details of large objects led us to investigate the possibility of using an X-ray detector with a smaller pixel size and with a greater number of conversion bits than what was used up to now. However, to use the apparatus already developed with both the detectors installed simultaneously the same X-ray source used for large objects was employed. The focal spot size of this source does not permit to reach the resolution achievable by means of a micro-focus X-ray source, but it could still be useful for some kinds of artworks. The capabilities of the experimental set-up were investigated directly on an artwork that is an ideal case to validate the method, i.e., a small wooden gilded Egyptian statuette from the Museo Egizio in Torino representing the goddess Taweret. One feature of this sculpture is the thin gold layer on the surface, presumably below 20 μm thick and visible despite its fragmentary state of preservation. It is worth noting that, to the best of our knowledge, few targeted studies have been focused on materials and techniques used in ancient Egypt for gilding, with a general lack of scientific data [23]. Therefore, a specific investigation with DR and CT analyses could be suitable to obtain information on the manufacturing techniques. 

## 2. Materials and Methods

### 2.1. The “Taweret” Statuette

**Figure 1:**
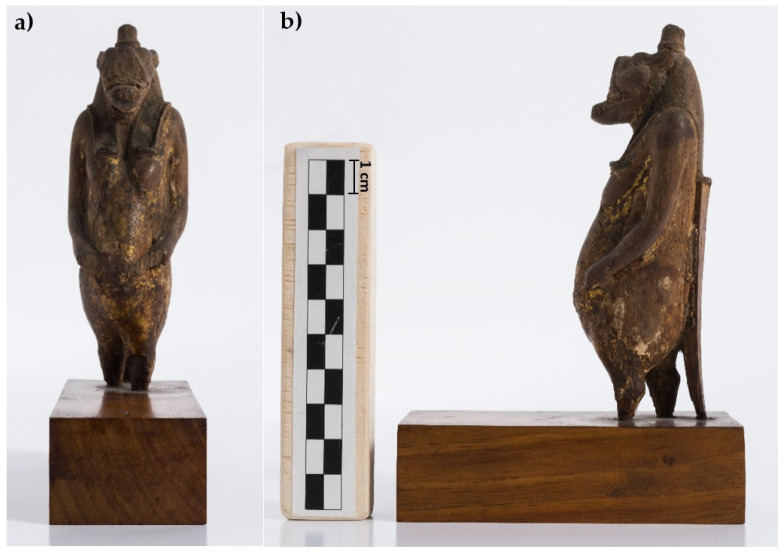
The analyzed Taweret statuette (Cat. 528), frontal (**a**) and lateral (**b**) views, before the conservation treatment.

The sculpture object of this study (height 12 cm × depth l4 cm × width 4 cm, Figure 1) belongs to the collection of Museo Egizio in Torino (Inventory Number Cat. 528). It is a representation of the ancient Egyptian goddess Taweret, whose name means “the great (female) one”. She is represented as a hippopotamus with human pendulous breasts, standing erect on its hind legs, the right one being slightly advanced. She has the tail of a crocodile and the legs of a lioness. She generally wears a tripartite wig often topped with a composite crown, a modio, or the sun disk. In most cases, the goddess Taweret handles a protection amulet (like “ankh”/life or “sa”/protection) [24]. In this statuette, the original wig, probably inlaid on the carved wood, is not preserved as it is for the crown and for any other symbol she could handle. The forward part of the muzzle being truncated, we cannot appreciate her fearsome appearance, with an open mouth exposing her tusks and tongue. The find is missing the feet and it shows evidence of fracture at the height of the ankles. Furthermore, the original base is lost, replaced by a modern one set up for exhibition functions. Being a purchased item (probably acquired with the Drovetti Collection in 1824), and giving the lack of any inscription, only some generic hypothesis about provenance, date, and function of the statuette Cat. 528 could be drawn. As far as we know, while stone and fayence amulets in the form of Taweret are well known from many settlements in Egypt, wooden statuettes depicting goddess Taweret are very rarely preserved. Two of them, probably from Deir el-Medina, are dated to the New Kingdom (1292–1119 BCE), but they could not be compared with Cat. 528 because they are very different in size and manufacture. Despite the difference in material, the sculpture object of this study could be instead stylistically compared with some statues of Taweret dated from the Third Intermediate to the Ptolemaic Period. As regards the function, Taweret was one of several goddesses who could take the shape of a hippopotamus. If the male animal was considered dangerous and destructive, the female animal was a manifestation of goddesses associated with the protection of childbirth and fertility [25]. 

They appear by the Old Kingdom (2625–2130 BCE) on amulets and on a variety of magical artifacts closely connected to pregnancy, birth, and care of newborns (magical wands, feeding cups, birth brick, scarabs, paddle dolls). Apotropaic images of Taweret also occur on household items such as beds, stools, and headrests but also on votive stelae. The statuettes of divinities could also be dedicated to the god and placed inside temples, particularly in the courts, in palaces, and in tombs. This statue, therefore, had a function linked to domestic worship, and it was most likely placed in a dedicated cult space inside the house or in a cult chapel inside a sanctuary. Several statues have been discovered in the Temple of Karnak and spectacular wooden gilded statues of gods have been found for example in the Tomb of Tutankhamon. 

**Figure 2:**
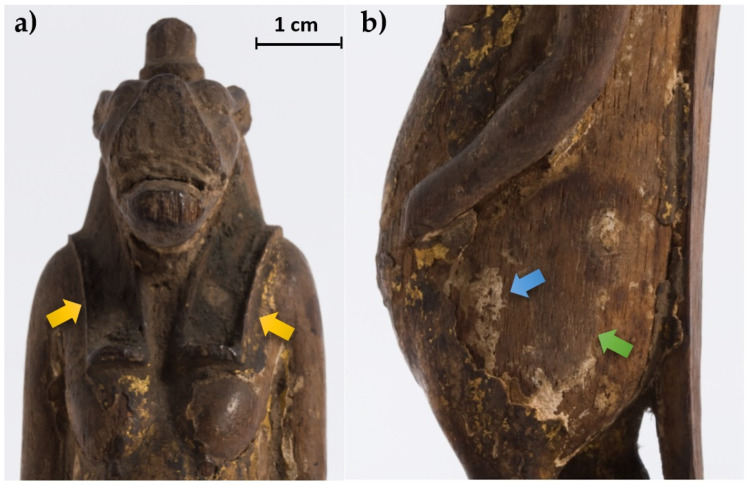
Some details of the statuette: (**a**) face and notches for the wig (yellow arrows); (**b**) detail of the body where the wood material (green arrow) and the preparation layer (blue arrow) are visible.

In relation to the manufacture, the sculpture is carved on wood and gilded with some colored details, features visible despite its fragmentary state of preservation (Figure 2). Gilding and inlaying finishing techniques were used on different items ranging as early as 3000 BCE to the Roman Period [26]. Gold had a symbolic and religious value linked to new life and gods, fictitious entities with golden skins. Different methods were used in ancient Egypt to gild organic (wood or cartonnage) and inorganic (plaster, stone, metal) substrates, gilding directly or on an additional substrate. After researching the literature on specific studies dedicated to gilding materials and techniques in Ancient Egypt and finding little information about the topic [27], it was decided to investigate the technique used for the decoration of this specific artifact through targeted scientific studies, among which was X-ray imaging.

### 2.2. Experimental

The X-ray imaging analysis was carried out at the CCR in a shielded 6 × 5 m^2^ room where the fixed apparatus developed in the framework of the neu_ART project is in operation. The system is composed mainly of a General Electric Eresco 42MF4 X-ray source, a rotating platform of about 1.6 m in diameter on which the object is placed allowing the acquisition of multi-angle images, and a linear X-ray detector [19,20,21]. The X-ray source has a focal spot size of 3 mm (EN12543) and a 900 W maximum power that means a maximum current of 4.5 mA at the highest voltage of 200 kV, increasing to a maximum of 10 mA at lower operating voltages. The detector employed is a 12-bit Hamamatsu C9750-20TCN line sensor camera with a 200 μm pixel dimension (but also 400 μm and 800 μm pixel dimensions can be selected via binning) and a length of 51.2 cm. The detector is installed on an x-y scanning system that allows us to acquire in a single scan a radiograph of up to 355 cm × 51.2 cm within a few minutes in real-time. Moreover, it is possible to carry out CT up to 2.5 m in height and 2 m in width. 

The system is suited for large objects DR and CT imaging, but the pixel dimension and A/D bit number of the detector limit the performances when small objects or details of large objects need to be investigated. Moreover, for small objects, the mechanical scanning system necessary when a linear detector is used takes a long time to carry out a CT compared to other methods, as for example the use of area detectors [2,3]. To overcome this problem, a new area detector was added and set up on the same x-y scanning system that hosts the linear detector. Specifically, a flat panel (FP) Shad-o-Box 6K HS by Teledyne Dalsa having a pixel size of 49.5 μm and a 14-bit A/D converter was employed. As the area is only 11.4 × 14.6 cm², this detector is more suitable for small objects or for parts of large objects where a higher resolution is needed. To cover large areas, it is possible to exploit the precision scanning system, already present, to acquire images in different positions (tile scanning). The main characteristics of the flat panel and the X-ray source are summarized in Table 1. The full capabilities in resolution (pixel dimension) are reached only if the analyzed object is placed near the detector. In particular, taking into account that the X-ray source is normally placed 350 cm far from the detectors, the penumbra is below 50 μm if the object is at a maximum of about 6 cm from it. This experimental condition can be reached in DR imaging for paintings on canvas and on wooden panels and for small objects, such as the Taweret statuette under investigation. 

In both DR and CT phases, the object has been positioned as close as possible to the detector to minimize the penumbra effect. The radiographs were acquired both frontally and laterally to have a more accurate view of the entire figurine. Due to the small size of the object compared with the active volume of the FP detector, it was not necessary to move the detector for radiographic and tomographic acquisitions. CT analysis was performed by setting the integration time and the angular step on all 360° in order to optimize the quality of the final reconstruction. The main experimental details of this measurement are summarized in Table 2. In the same table, the experimental conditions from three previous CTs obtained by means of the linear detector are also shown [10,21,22]. From the comparison, it is possible to observe the better voxel dimension, 46 μm, limited only by the penumbra. In fact, due to the base that is larger than the body of the statuette, it was not possible to place the object at less than 6 cm from the detector. In any case, the distance of 24 cm is lower than what is achievable by means of the linear detector. The apparatus was preliminarily tested on another wooden statuette without gilding [28]. 

The reconstruction of the CT sections was made using a filtered back-projection algorithm [29,30] (fan-beam geometry) by means of a non-commercial software-utility developed by Dan Schneberk of the Lawrence Livermore National Laboratory (USA), while the 3D rendering and segmentation were realized using VGStudio MAX 2.2 from Volume Graphics.

## 3. Results and Discussion

At first observation, the statuette appears carved from a single solid wood, whose material is partially exposed due to widespread gaps in the surface decoration. The wood appears quite dark and very compact. 

**Figure 3:**
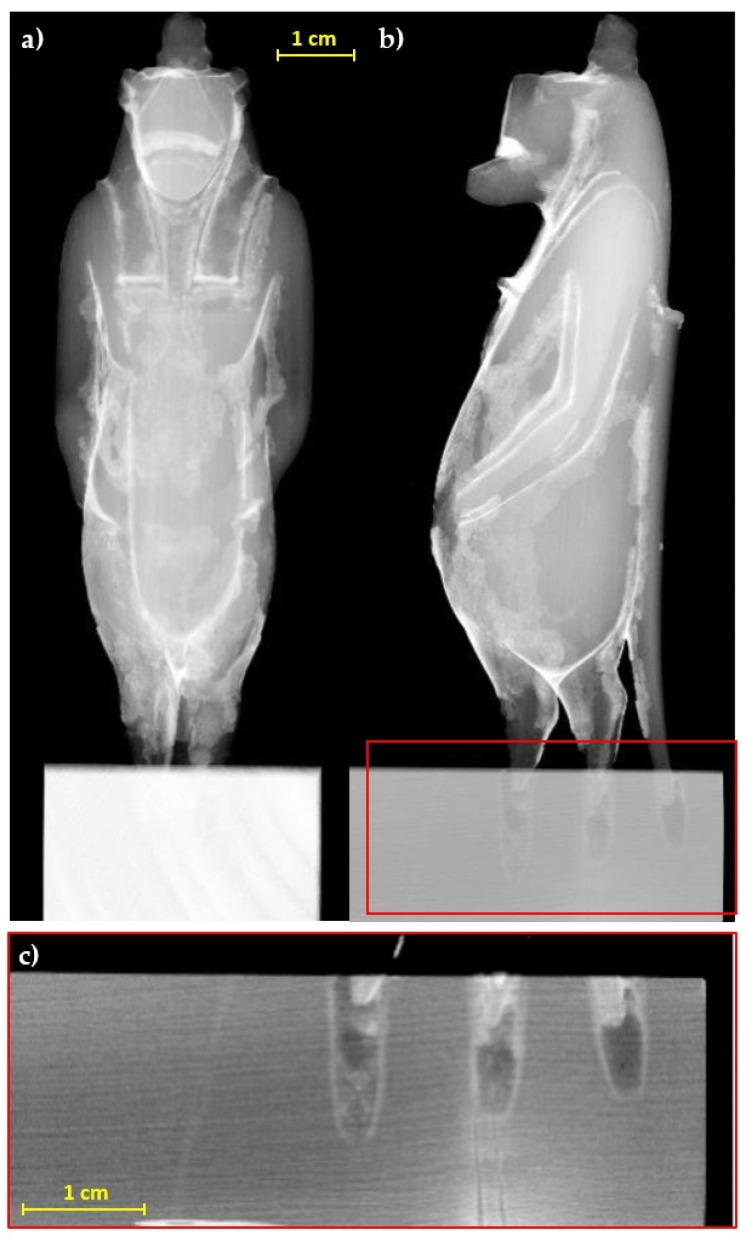
Radiographs of the statuette, (**a**) frontal and (**b**) lateral view; (**c**) detail of the legs and tail insertion in the modern base.

The radiographic analysis (Figure 3), particularly useful for an initial assessment of the state of preservation, allows observing the portion of the legs and tail inside the base, which are inserted for about 3–4 mm. Furthermore, regarding the structural stability of the decoration layers, the radiography allows highlighting diffuse detachments of the preparation from the support (not visible to the direct observation), slightly darker than the response of the preparation layer, even in correspondence with well-preserved areas.

**Figure 4:**
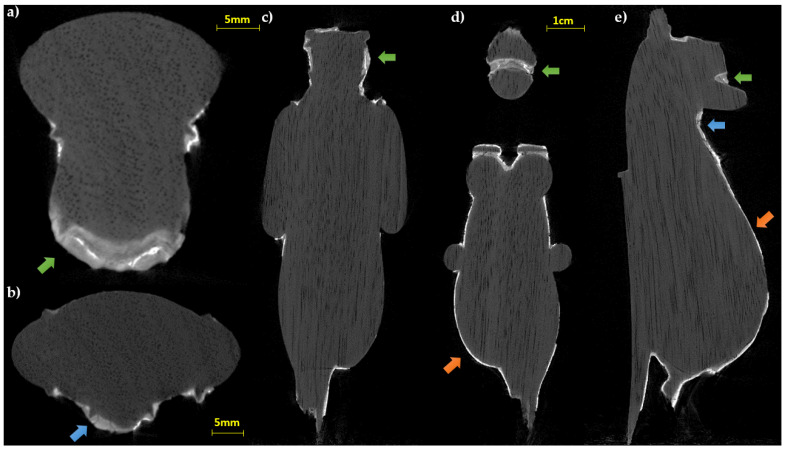
CT slices of the statuette: (**a**,**b**) horizontal sections; (**c**–**e**) longitudinal slices. In all the sections the wood structure (vessels and growing rings) is visible (green arrows: preparation layer in the face area; blue arrows: fractures inside the preparation layer; orange arrows: thin preparation material for the gold leaf adhesion).

The evaluation of the horizontal (in which the vessels of the wood are clearly visible, see Figure 4a,b) and longitudinal slices (Figure 4c–e) obtained from the CT analysis confirm the first visual observation and allow us to hypothesize the use of a sub tangential cut taking into account the position of the growth rings. 

As for the subsequent manufacturing phases, it can be observed that a preparatory layer has been applied to the wooden support. The stratigraphy, analyzed in detail thanks to an erratic sample of gilding with other techniques (optical microscopy, infrared spectroscopy, and scanning electron microscopy), has confirmed the presence of a thick white preparatory layer composed of calcium carbonate and an organic substance (a mix between a protein compound and probably a natural resin).

This was functional to the application of the metal leaf and presumably also useful for definitive modeling, taking into account the greater thickness of the preparatory layer in some parts of the body, which are visible also to the naked eye. 

**Figure 5:**
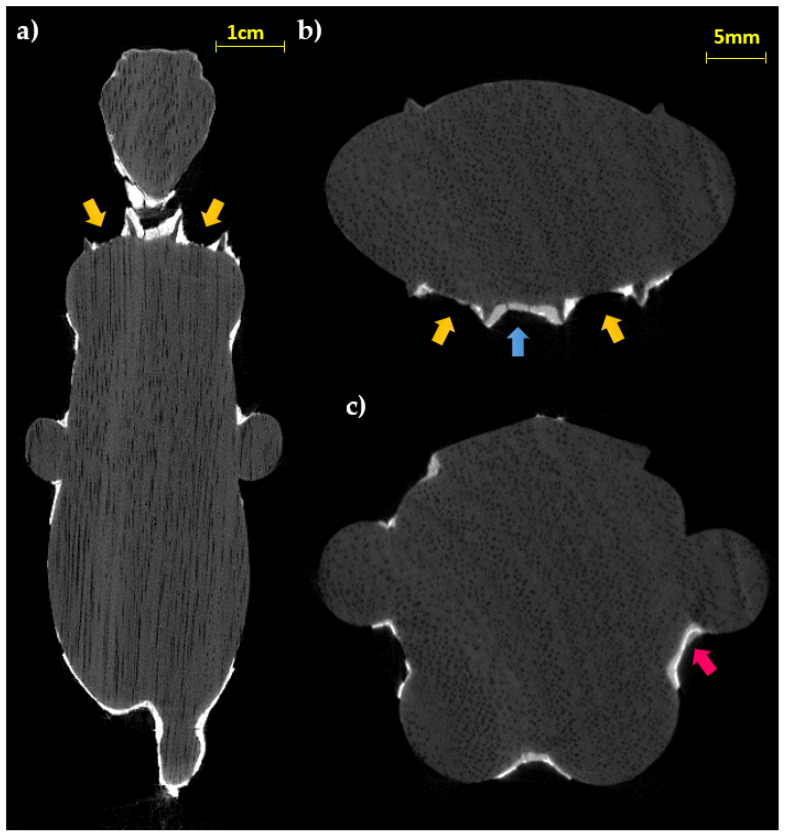
CT longitudinal (**a**) and horizontal (**b,c**) slices (yellow arrows: wing grooves; blue arrow: fractures inside the preparation layer; pink arrow: material layer above the gold leaf).

**Figure 6:**
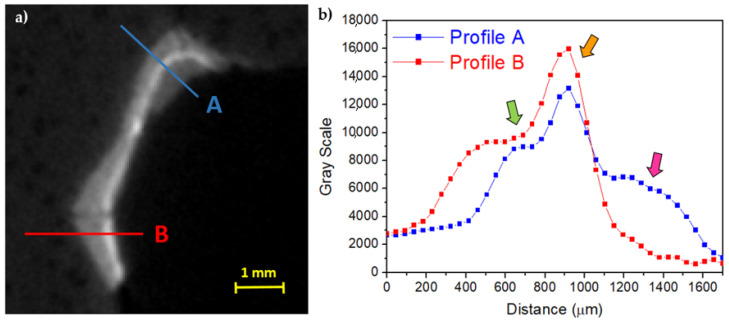
(**a**) Enlargement of Figure 5c and (**b**) the profile plots along A and B (green arrow: preparation layer; orange arrow: preparation material for the gold leaf adhesion; pink arrow: material layer above the gold leaf).

The thicknesses of the preparation layer in those areas, beneath the gilding, can be observed in the horizontal slices of the CT scan (Figure 4). Especially in some areas, the preparatory layer is of great thickness, such as, for example, in correspondence of the truncated muzzle for which a more conspicuous modeling intervention is therefore hypothesized. In some places, it is also possible to observe micro-fractures inside the preparation layer itself (Figure 4b,e). On the surface, it is possible to visualize limited areas with high radiopacity, above the preparation, presumably identifiable with the applied gold leaf, and in some places, with another layer of lower radiopacity material placed above (similar to that of the preparatory layer, but slightly less radiopaque), attributable to a blackish-brown material visible to the naked eye (partially composed of a protein substance and a fat substance or natural resin, with a heterogeneous deposit of calcium carbonate, gypsum, and silicates, detected by Infrared Spectroscopy (FT-IR) analysis, Figure 5c). In Figure 6, an enlargement of Figure 5c and the profile plots in two regions of interest are shown. It is possible to better observe the different thicknesses of the preparation layers in the two parts of the object (thicker in part B than in part A), the peak due to the gold layer, and the contribution of the blackish-brown material (present only in part A). 

The observation of the slices at the height of the terminal part of the wig (Figure 5a,b), probably carved in the wooden material for the application of a relief decoration, allows us to appreciate both the regularity of the grooves and to confirm the absence of the preparation layer inside; in fact, upon direct observation, it shows traces of color but not of preparation. This data can confirm the hypothesis of the direct application of another type of material (e.g., relief decoration or frit), as already found in other finds from the same period [31]. Instead, stratigraphy like that of the body (preparation layer with the metal leaf applied on it) is found in the area of the sternum (Figure 5b,c). 

**Figure 7:**
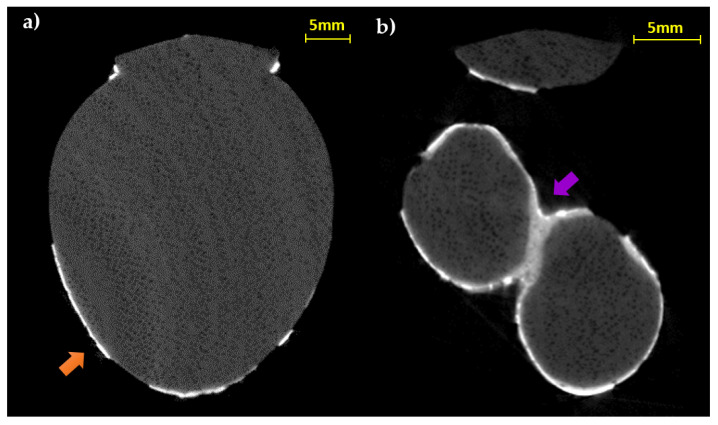
CT horizontal slices of the body (**a**) and the legs (**b**) (orange arrow: thin preparation material for the gold leaf adhesion; purple arrow: thick preparation material between the legs).

The slices corresponding to the body confirm a rather homogeneous distribution and thin thickness of the preparatory layer (Figure 7a), confirming, in this case, the application of this material for the metal leaf adhesion and not for modeling the object. Only in correspondence with limited notch defects, does the layer have a shape corrective function. Additionally, the tomographic analysis of the terminal portions (legs and tail) allows verifying the presence of the decoration even in correspondence with the less visible portions and between the legs (Figure 7b).

**Figure 8:**
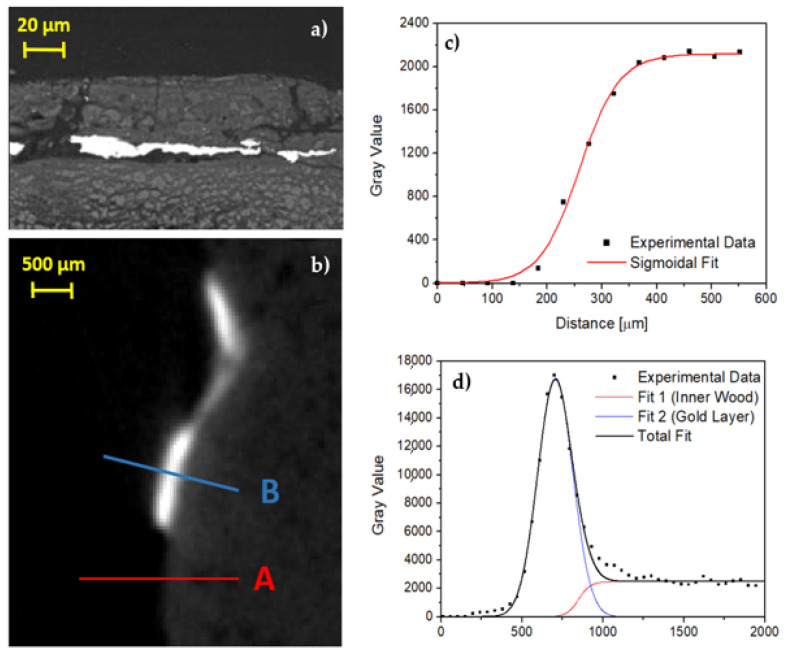
(**a**) Backscattering image by means of SEM, the gold layer is in light gray; (**b**) enlargement of Figure 5c; (**c**) profile plot along A line shown in (**b**), only wood; (**d**) profile plot along B line shown in (**b**), gold on thin preparation layer.

Regarding the gold layer, analysis on two erratic fragments through scanning electron microscopy (SEM) was carried out, resulting in 5–10 μm thickness (Figure 8a) in accordance with previous literature [27,32]. Despite the gold layer being clearly observable by means of CT, the thickness is smaller than the voxel size (46 μm) and the penumbra due to the measurement geometry (205 μm). In any case, an attempt to extrapolate some information about the thickness of the gold leaf was made, in order to test the methodology used and to verify the maximum resolution obtainable in such experimental conditions. In Figure 8b an enlarged area of Figure 5c is shown. The area was selected because there are parts with gold on a very thin preparation layer and parts without any surface layer, i.e., the wood is exposed. A first fit using a sigmoidal curve was made on this last part and the results, shown in Figure 8c, were used as starting parameters for the wooden part of the profile shown in Figure 8d and related to the gold layer. In this second profile, the contribution from the preparation layer is negligible and it produces only a very small band on the right of the gold peak. In this way, using a first approximation gaussian fit for the gold layer, a value of 2σ = 216 ± 7 μm was obtained that confirms the limits due to the geometrical conditions used.

**Figure 9:**
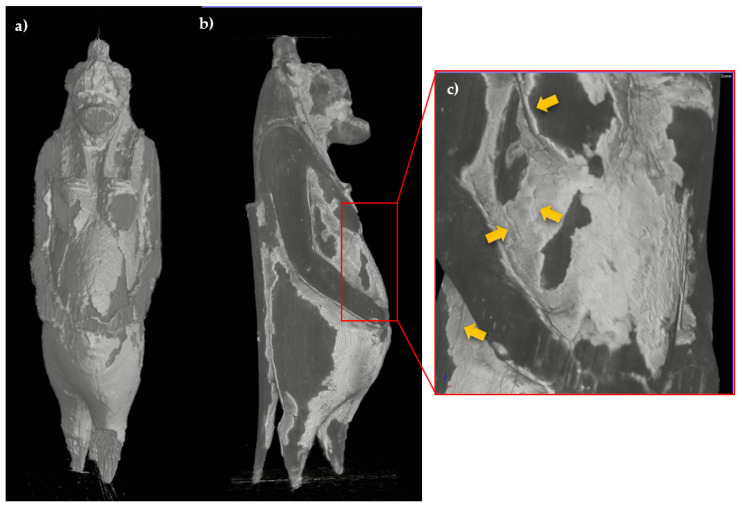
3D model of the statuette with different surface render tools, ((**a**), Scatter Gradient v1.2 tool), ((**b**), Hardware render (scatter gradient) tool); enlargement of the body area in which the cracking is visible (yellow arrows) (**c**).

**Figure 10:**
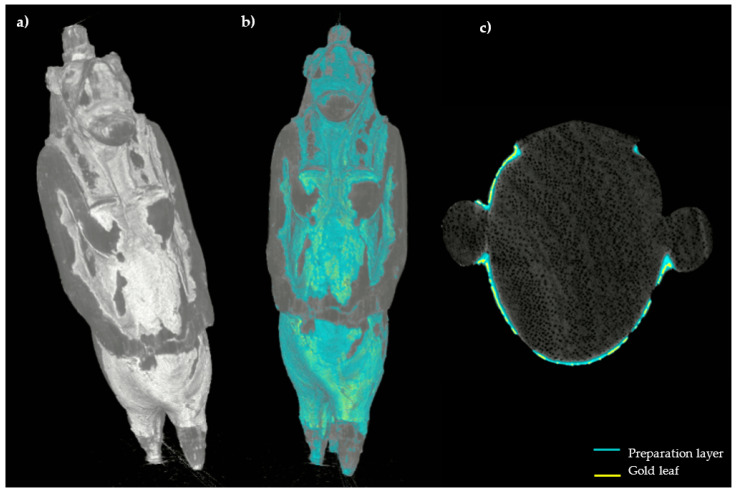
3D model of the statuette ((**a**), Hardware render (scatter gradient) tool) with the segmentation performed on the surface (**b**); CT horizontal slice in which the segmentation results are highlighted (**c**).

Finally, the 3D model of the statuette was created from the CT horizontal slices: different types of surface render tools were used to highlight the distribution of the preserved decoration boosting the rendering of its specific superficial features, see Figure 9. Here it is also possible to notice some diffuse cracking on the layer above the wood. Then, an attempt of segmentation based on the different grey level responses of the preparatory layer and the gold leaf was performed both on the 3D model and in the horizontal slices, in order to better visualize the distribution of the used materials. The results are visible in Figure 10, in which the blue color corresponds to the preparation and the yellow to the gold layer.

## 4. Conclusions

The applications on Cultural Heritage of digital radiography and computed tomography with X-rays allow non-destructive analysis for the diagnostics of archaeological objects and artifacts and, most often, require the use of ad hoc and versatile X-ray imaging systems. This is the case of the instrument working at the Centro Conservazione e Restauro “La Venaria Reale” near Torino, in Italy, that was developed in collaboration with the National Institute for Nuclear Physics (INFN) and the Physics Department of the University of Torino. The DR and CT analysis carried out for the study of the Taweret wooden statuette of the Museo Egizio in Torino were performed thanks to an upgrade of the existing set-up. In fact, a flat panel detector with higher resolution compared to the linear detector already present in the X-ray imaging laboratory was used: with a single-pixel size of 49.5 μm, it is possible to visualize finer details and obtain more precise information with respect to the previous apparatus, particularly for small objects. 

Radiographic and especially tomographic analysis of the Taweret statuette highlighted its inner wooden structure, confirming the hypothesis of its making from a single block. Furthermore, the presence of a preparation layer material was verified according to the application of the gold leaf, placed as a decoration of the artifact, and in some places for the modeling of the final shape. This is confirmed by the variable thicknesses found in the preparation layer of the statuette. Moreover, in the grooves made for the wig insertion, the absence of the preparation layer would confirm the hypothesis of a possible application of another type of material for hair realization. Finally, a 3D model of the statuette was created in which the preparation and the gold layer were highlighted.

## Figures and Tables

**Table 1:** Technical specifications of the main instrumentation used for the analysis.

Detector: FP Shad-o-Box 6K HS		Source: GE 42MF4	
Pixel number	2304 × 2940	Target	tungsten
Active area	11.4 × 14.6 cm^2^	Voltage	5–200 kV
Pixel size	49.5 μm	Max. Current	10 mA
A/D converter	14 bit	Max. Power	900 W
Energy range	15–225 keV	Focal spot (EN12543)	3 mm
Scintillator	CsI	Beam angle	40 × 60°
Data transfer	Gigabit Ethernet	Exit window	0.8 ± 0.1 mm, Be

**Table 2:** Experimental CT conditions.

	Furniture [21]	Coffin [10]	Soil block [22]	Taweret
Dimension of the object	129 × 59 × 312 cm^3^	31 × 50 × 182 cm^3^	10 × 15 × 40 cm^3^	4 × 12 × 14 cm^3^
X-ray tube voltage	180 kV	180 kV	200 kV	80 kV
X-ray tube current	5 mA	5 mA	4.5 mA	10 mA
X-ray filter ^(a)^	Al (2 mm)	Al (2 mm)	Al (2 mm)	Al (2 mm)
Source-Detector Distance (SDD)	295 cm	369 cm	294 cm	375 cm
Source-Object Distance (SOD)	214 cm	318 cm	264 cm	351 cm
Object-Detector Distance (ODD)	81 cm	51 cm	30 cm	24 cm
Magnification	1.38×	1.16×	1.11×	1.07×
Dimensions of a projection ^(b)^	210 × 51.2 cm^2^	90 × 51.2 cm^2^	26 × 51.2 cm^2^	11.4 × 14.6 cm^2^
Area of a projection	1.07 m^2^	0.46 m^2^	0.13 m^2^	0.017 m^2^
Detector scan speed	5.0 m/min	2.2 m/min	2.0 m/min	Fixed
Integration time per Pixel	9.6 ms	10.9 ms	6.0 ms	1.75 s
Number of projections	720	1080	540	1440
Acquisition time for a projection	25.2 s	24.5 s	7.8 s	1.75 s
Total acquisition time ^(c)^	10 h	15 h	3 h 40 min	1 h 55 min
Detector pixel size	200 μm	200 μm	200 μm	49.5 μm
Pixel dimension ^(d)^	800 μm	400 μm	200 μm	49.5 μm
Reconstructed voxel size	580 μm	345 μm	180 μm	46 μm
Penumbra	1.14 mm	480 μm	340 μm	205 μm
Dimension of one projection	51.3 Mb	22.0 Mb	6.3 Mb	12.9 Mb

^(a)^ The use of an aluminum filter avoid the beam hardening effect; ^(b)^ for objects higher than the detector length more than one portion is acquired: here the parameters for one portion are reported; ^(c)^ the total time is related to a portion (also the dead time due to the linear scan, the rotation stage movement and the source cooling that is periodically switched off are taken in account); ^(d)^ in some cases the pixel dimension of radiographs is higher than the detector pixel size because of the binning (2 × 2 or 4 × 4).

## Data Availability

The data presented in this article are available on request from the corresponding author.
